# The Galperin–Talyzina Method of Psychological Investigation of the Genesis of Cognitive Processes

**DOI:** 10.11621/pir.2023.0304

**Published:** 2023-09-30

**Authors:** Andréa Maturano Longarezi, Iury Kesley Marques de Oliveira Martins

**Affiliations:** a Uberlandia Federal University, Uberlandia, Brazil; b Goias Federal University, Goiania, Brazil

**Keywords:** cultural-historical psychology, developmental didactics, experimental method, gradual formation experiment, P.Ya. Galperin, N.F. Talyzina

## Abstract

**Background:**

Piotr Ya. Galperin and his collaborator Nina F. Talyzina performed solid experimental work which led them to propose the theory of stage-by-stage formation of mental actions and concepts, as well as a method to investigate cognitive processes, whose conceptual and procedural streamlining demands analysis and systematization.

**Objective:**

To investigate the formative experiment of P.Ya. Galperin and N.F. Talyzina, with the aim of analyzing their contributions to the method of psychological investigation of cognitive processes.

**Design:**

The article is part of a theoretical research project on developmental didactics systems, of which the Galperin–Talyzina system is one. Russian works by the two authors and their translations into English, Spanish, and Portuguese, as well as works by other psychologists and educators from the Galperin–Talyzina school, were sources of the work.

**Results:**

The experiments of P.Ya. Galperin and N.F. Talyzina studied, promoted, and analyzed the assimilation of new knowledge and mental actions, by introducing different conditions. The article systematizes the stages and procedures of such experiments, as well as the series and steps of diagnosis of the developmental level and the formation of mental actions and concepts, in relation to the type of Orienting Basis of an Action (OBA).

**Conclusion:**

The theory was built on the basis of simultaneous production of a method to study the genesis of the cognitive processes and the theory of stage-by-stage formation of mental actions and concepts; it led us to define the method of P.Ya. Galperin and N.F. Talyzina as a gradual formation experiment.

## Introduction

Piotr Ya. Galperin (1902–1988) performed solid experimental work in the methodology of psychological investigation, and was internationally recognized. Nina F. Talyzina (1923-2018) effectively participated in the ongoing experimental activity and consolidated herself as a prominent collaborator of the system. The contributions of their work are not limited to the proposal of a theory; above all, they include a method for investigation in the area of psychology.

Soviet, Russian, and international psychological literature recognize Galperin as an important contributor to Marxist psychology, with special attention to the genesis of cognitive processes. A bibliographic review in the field allows us to highlight the relevance of Galperin’s work (Arievitch & Haenen, 2005; Haenen, 1988, 1993; Karpov, 1983; León, 2019; Longarezi, 2020a, 2020b, 2021a, 2021b; Núnez, Pinheiro, & Gonçalves, 2019; Puentes & Longarezi, 2020; Solovieva, 2014a, 2014b, 2014c; Solovieva & Rojas, 2020; Talizina, Solovieva, & Quitanar Rojas, 2017). Galperin opened up a new object of study for psychology (orientation as the primordial function of the psyche), proposed a theoretical approach for this object (the stage-by-stage formation of mental actions), and designed a method for its apprehension (the gradual formation experiment, as we have named it).

Galperin and his collaborators produced over 800 experimental works (Galperin, 1950, 1957 [2001b], 1957 [2001d], 1959 [2001c], 1959 [2017], 1959, 1965 [2001a], 1965, 1976 [1992a], 1977 [1992b], 1977 [1992c], 1978 [1992d], etc.), and he stands out as an important contributor to the theoretical framework of Soviet psychology and didactics, including a new perspective that made him a pioneer, not only for his theoretical collaboration, but also for his determination of “orientation as a fundamental function of psyche” as an object of psychology, which constitutes an essential element in all his theoretical and methodological propositions.

Galperin’s importance was recognized by several important representatives of Soviet psychology (A.V. Zaporozhets, A.R. Luria, A.N. Leontiev, D.B. Elkonin, V.V. Davydov, and V.P. Zinchenko, among others). Our bibliographic review of the experiment inaugurated and developed by the author to building the Galperin–Talyzina^1^ system, gives visibility to the deepness, amplitude, and consistency of the experimental work of Galperin and his collaborators. Moreover, it is important to highlight the impact of his theoretical and scientific enterprise on the psychological theory of that period.

Galperin worked along with several researchers and groups, since his entrance to the Kharkov school in 1930, his entrance to Moscow State University in 1943, and his experimental work after the promulgation, in 1958, of the resolution “*On the work of the Pedagogical Science Academy of the Transcaucasian Soviet Federative Socialist Republic and on the tightening of its bonds with the schools and centers of pedagogical investigation.*”

Before considering the relevance of the author’s contributions to psychological investigation and developmental didactics, we look at the experimental method which was produced along with Galperin’s gradual formation theory^2^ as an object in the current article, which has as a focus the contributions of historical-cultural psychological investigation.

## Method

The current work analyzes the formative experiment in the Galperin–Talyzina system, highlighting the main contributions made to the method of psychological investigation of cognitive processes. The study consists of theoretical research, for which a significant part of the author’s work was consulted (Galperin, 1950, 1959, 1965, 1966, 1979, 1983, 1976 [1992a], 1977 [1992b], 1977 [1992c], 1978 [1992d], 1965 [2001a], 1957 [2001b], 1959 [2001c], 1957 [2001d], 1959 [2001e], 1959 [2001f]). Works dealing mainly with the research method of the Galperin–Talyzina didactic system were selected, as well as those about the theory of stage-by-stage formation. In addition to monographic works and scientific studies by psychologists and educators who worked with him or in parallel to him, we reviewed the work of critics and researchers of the Galperin school. The theoretical research was based on the original sources in Russian, or translations into English, Spanish, and Portuguese. Analysis and synthesis produced in the investigation followed a documental research process, searching for information and interpretative meanings, “…the path and movement tracked in the building of the study were to identify, select, interpret, produce ‘pieces’ and the way of best fitting them together to construct an understanding of what has been produced about the experiment” (Longarezi, 2019, p. 169). We emphasize that this work is part of a major study of developmental didactic systems, of which the Galperin–Talyzina system is one. Therefore, the research problem was to characterize the research method produced by the group coordinated by Galperin as a starting point for future studies of the similarities and differences of experimental work carried out by representatives of different developmental didactic systems.

## Results and Discussion: Study of the Genesis of Cognitive Processes

The great academic and scientific contribution of P.Ya. Galperin resulted in studies that led to a method of investigating the genesis of cognitive processes, *the theory of stage-by-stage formation of mental actions* (Galperin, 1966, 1976 [1979]). Galperin's career started in the 1930s and the experimental programs in education took place in the 1950s; the elaboration of the theory took place especially from 1940 to 1970, focusing the author’s theoretical thought on children approximately 6 to 11 years old, an age when the main activity is study.

Among the aims of the method, three are specifically remarkable: 1. to define the stages of the formation of concepts and mental actions, with a focus on the psychological orientation of activity; 2. to defend the inseparability of knowledge and ability in this formation process, showing that concept formation takes place in unity with the formation of mental actions; and 3. to create the conditions for theoretical thought to develop between the ages of 5 and 6 years, through the mediation of the object in its material or materialized form.

Galperin announced his theory in 1952, presenting it as “a hypothesis of the formation of mental actions,” at the first of three conferences of the All-Union Experimental Medicine Institute, which marked the “rebirth of psychology,” after the decree of 1936, which put an end to the positions and responsibilities of paedologists at schools and banned paedology as a university subject. Not originally developed as a pedagogical theory, it was conceived as “a theory of the ontogenetic formation of psychic activity” (Talizina, 1988, p. 137); as this ontogeny is an assimilation process in collaboration with other people, it has been, simultaneously, a didactic process; thus: “this theory is, at the same time, an assimilation theory, a learning theory” (Talizina, 1988, p. 137).

The basis for the creation of such a theory is in the understanding of the existing genesis relationship between mental operations and external practical actions. This suggests that a child’s thought develops through a connection with objective activity (the direct handling of objects). Galperin investigated the conditions of transformation of an external action (in a gradual form) into an inner action, going through a series of successive stages, each one forming the basis for the following stage; this was the general principle of his method and followed him in the formative experiments or gradual ones that he performed.

From this perspective, the method and theory predict the disclosure of the inner structure of the action, inaccessible to direct external observation, but existing objectively (Shabel’nikov, 2012); and reveals the mechanisms, laws, and conditions for the formation of the constitutive aspects of mental activity. Along with the research done by Galperin and his collaborators about the stage-by-stage formation of mental actions and concepts, there is also a need for an assessment of the quality of the formed action.

### The Gradual Formation Experiment: Conception, Stages, and Methodology

Galperin proposed an investigative method of the formative kind, which we have named “a gradual formation experiment” (Longarezi, 2021a; 2021b), and which has also been named “a systematic experiment” (Haenen, 1993), because the process is “a systematic formation” (Galperin, 1983; Haenen, 1993), which is the same as “the stage-by-stage formation of mental actions and conceptions.”

The genesis of such studies is connected to the history of the formative experiment, the methodology used in the context of historical-cultural psychology since the initial works of L.S. Vygotsky [transliterated in Portuguese as Vygotsky in the bibliography and citations] (1896–1934), with the genesis-cause method; with later investigations by A.N. Leontiev (1903–1979), A.R. Luria (1902–1977), A.V. Zaporozhets (1905–1981), L.V. Zankov (1901–1977), P.Ya. Galperin (1902–1988), D.B. Elkonin (1904–1984), V.V. Davydov (1930–1998), V.V. Repkin (1927), and their groups of collaborators.

From the experimental perspective developed by Galperin, the experiment targets the formation of scientific thought in the child, and the conditions that promote the formation of mental actions. We may understand the formative experiment, under the perspective assumed by Galperin, as a work of intervention which studies, promotes, and analyzes the “assimilation of knowledge and its new actions during the introduction of different conditions in the process of its formation” (Talizina, 2000, p. 29). The formative experiment consists, therefore, in a process of psychological-pedagogical intervention which aims at studying the formation and development processes of mental actions; it consists, thus, of the developmental process of learning during stages that permit the formation of mental actions.

The formative experiments predict the following steps: “1. delimitation of the objective: specifying a hypothesis in a determined task; 2. planning of the experimental steps; 3. the experiment itself: obtaining of data; 4. data analysis; and 5. the conclusions which the data may permit us to reach” (Talizina, 2000, p. 27).

The methodology of the gradual formation experiment, in turn, includes: explanation for the subjects of the components of the concept; presentation of such components on cards; presentation of the determination of the phenomenon as a drawing or in written form; explanation by the subject of each of the elements from the drawing or the phrase; repetition of the operation, aloud, without using the cards; application of the concept in all its possibilities, thus creating the conditions for its generalization; and repetition of the actions at different levels (Galperin, 1957 [2001b]).

### Diagnosis of the Developmental Level and the Formation of Mental Actions and Concepts

In the experiments by Galperin, Talyzina, and their collaborators, the stages of the child’s intellectual development were diagnosed by using different forms of the Orienting Basis of an Action (OBA)^3^ (concrete, perceptual/through images, and/or logical-verbal), which permitted, beyond the identification of acceptability in collaboration, the delimitation of the developmental level of the student, that is, the potential of their developmental zone: “... the formation experiment (for diagnosis) rests on the zone of proximal development, in which the psychologist builds (forms) in the child the action or the new habit. Therefore, the use of the zone of proximal development for diagnosis of the intellect, requires the selection of actions (tasks) that are new (unknown) and, at the same time, accessible to the child; that is, they are found in the child’s zone of proximal development. During the formation of the new intellectual action, the psychologist helps the child as an orienting basis of an action, providing the kind of support the child needs; the child’s work on that basis shows whether the child is able to accept help or not, whether the help has been effective or not. In other words, the concept of an orienting basis of an action leads to an understanding of the support which the psychologist gives the child during the formative experiment” (Solovieva, 2014a, p. 148).

In the experiments, children were presented with new intellectual actions, with problems to solve. This process presumes two stages. In the first, an OBA is presented on the concrete plane, so that the possibilities of doing it on the logical-verbal plane, with concrete images and concrete actions, may be investigated. The second stage presumes providing an OBA on the logical-verbal plane, to later raise the possibilities of an independent execution on the same plane. If it is not developed on this plane, it is presented in the form of images, and if a solution is still not possible, it is finally presented in concrete form.

In the experimental process, if the objective is diagnostic, it is important that the movement be from the upper plane (logical-verbal) to the lower plane (concrete), and if the intention is to form actions as an educational process, the movement should be in the opposite direction.

Actions do not need to be kept in execution; that is, it is possible to suppress some of them if the child executes the action on the upper level, the logical-verbal plane; that means that the child does not need to go through all the actions (of lower levels). The logical-verbal is, therefore, the first “indicator of the qualitative and global criterion of the child’s developmental level (Karpov, 1983)” (Solovieva, 2014a, p. 151). The second indicator is the success of the activity mediated by OBA. If the child does not perform the action at a logical-verbal level, they are advised to try it using the OBA scheme provided. “If the first criterion is related to the intellectual development plane on which the child performs the action, the second criterion is related to the intellectual development plane on which the child accepts the Orienting Basis of an Action, which is provided by the psychologist” (Solovieva, 2014a, p. 151).

These experiments revealed that the highest plane on which the child accepts the OBA scheme signals that the child’s development corresponds to the highest plane on which he or she performs the new action. In the studies of intellectual development, the experiments assume two indicators and/or criteria, which also points out two ways of assessing the developmental planes: the execution plane and acceptance of the adult’s explanation of the scheme.

The experiments reveal the correlation between the level of generalization and consciousness, if the plane of intellectual development is on the logical-formal plane. That means that the experiments done by Galperin and his collaborators establish a relationship of dependence between the characteristics of the action and its form (Le, Van An, 1995).

Investigations, not restricted to the former Soviet Union like the works done in Moscow (Talyzina & Karpov, 1987; Karpov, 1983), and including studies in Vietnam (Le, Van An, 1995) and China (Chzhao Hunchzhun, 1995), revealed methods of psychological diagnosis whose data demonstrated that success in the formation of scientific concepts does not depend only on chronological age (the periodization of the child development process), but also on the child’s intellectual development plane (Solovieva, 2014a; Zaporozhets, 1996).

The experimental method for diagnosis and the formation of the concept and mental action includes, at least, two series to be described below, so that the reader understands the collaborative movement of the subject in relationship with the other students, as well as the place of OBA in the whole experimental process. Let us go to the first series of the study.

### The First Series of the Experiment

The first series of the experiment for assessment of the level of intellectual development includes three stages: 1. *identification of the newness of the action*; 2*. presenting of OBA;* and 3. *checking the intellectual development level in the Possible Development Zone (PDZ)* (Solovieva, 2014a).

Stage 1 consists of *identifying the newness of the action.* The known action may be assessed from other characteristics of the action, be it the level of automatization, reflection, assimilation, reduction, or even generalization. However, concerning the assessment of the newness of the action, this is presented at a lower level, on the plane of concrete actions. If the child does not solve the problem on this plane, it means that the action is new to that child. If the child solves the problem and one intends to verify the level of assimilation of the action, the stage may include verifying it at the logical-verbal level. The experimenter may require only identification of whether the action is new or not, but may want to delimit the level of assimilation, on the verbal, perceptive, or concrete plane.

To check those, verbal problems are presented. If they are solved (without collaboration) one may conclude “that the assimilation plane of such action was logical-verbal and that this child already has the orienting basis of an action enough to solve this intellectual action in an independent way” (Solovieva, 2014b, p. 187). That means this child’s action is on a logical-verbal plane, in the child’s PDZ. For these cases, checking the newness of the action ends in the first series of the experiment and we proceed to the second. If the child does not solve one of the problems, the object is presented in a material form. If both are solved, it means that the level of development is on the plane of concrete images. If one of the problems is not solved, the level is on the plane of concrete actions. From the conclusions of this stage, we proceed to the second series of the experiment.

To sum up, from *checking the newness of the action,* if the action is new, we proceed to the following stage of OBA; if the opposite happens, the child is presented with problems at a verbal level; if the problem is still not solved, it is presented in the form of images.

Stage 2 comprehends the *presentation of OBA* and it starts as soon as the newness of the action is verified and also its respective level of assimilation, when it composes part of the experiment. When one is working on the first series of the experiment, the OBA scheme is presented on the plane of concrete actions and it takes place at different levels of collaboration, from a more rudimentary level of OBA to its complete scheme.

If after the explanation of the problem the child quickly solves it, that means that the child has assimilated the scheme at a lower level of support^4^, the first level. When the problem is solved with only the first level of support, the psychologist experimenter starts verifying it in his plan of independent execution and, if there is no success, proceeds to the second level. In general, a calculation is performed of the percentage of children who solve the problem at the first level in different social classes. At the second level, greater support is offered, although it does not constitute the complete level of orientation. At that level, the experimenter repeats the procedure adopted in the first level; however, support goes a little further. Explanations are followed by concrete actions, so that by offering support, in a complete way or not, the experimenter keeps creating the conditions for the child to solve the problem independently. This process takes place as long as it makes it possible for the child to solve the problem. If a correct solution is found from this second level of support, the child is instructed to solve the problem independently. Once solved, the identification of the upper plane of independent execution is followed. However, if success has not been achieved for this resolution, the psychologist proceeds to the third level of support.

The third level consists of a complete explanation, using all available procedures to solve the problem. The explanation, followed by a concrete demonstration, is open to the manipulation of objects, from which observation, comparison, and the establishment of relationships take place. Manipulation is held at this level, in the most detailed form, because the experimenter points out all the elementary actions so that the problem may be solved. That is why the third level represents the complete scheme of OBA. Only after solving the problem, with support at this level, is the independent execution plan followed.

The aim of this stage of *the presentation of OBA* is to lead the child to understand the principle of the execution of the action, its logic; if the child does not solve the problem, he or she accepts the adult’s explanation and copies it. If the child is not successful at one of these levels, it means that the adult may have made some procedural mistake.

Finally, stage 3 comprehends *verifying the intellectual level at the PDZ.* It is expected here that the child will solve the problem, and the independent execution may be observed from the upper plane of intellectual development. Thus, the problem is presented on the logical-verbal plane, perceptive and material, as explained, by the teacher to the child.

### The Second Series of the Experiment

In the second series of the experiment, another problem is introduced, called “third exclusion” or “elementary classification” (Solovieva, 2014a). What distinguishes this second series from the first one is that the OBA is present simultaneously on the three planes of intellectual development: logical-verbal, perceptive, and material. For each orienting basis, the solution to the problems needs to take place at the level presented.

Since the probability of aleatory responses is higher in this second series of the experiment, two types of problems are used. To verify the newness of the action, they are presented on the material plane. Just as for the first series of the experiment, if the child does not solve them, that is because they are new; if not, the experimenter investigates their solution on the perceptive or logical-verbal level. The solution to the problem on these two planes points out that their real level is on the verbal plane of assimilation. But if at least one of them has not been solved, two other problems on perceptive plane are presented. The solution of both confirms the level of development on this plane; if this is not the result, the developmental level is on the material plane.

The difference in the elaboration of the scheme of OBA in this second series is that it is presented on the three planes simultaneously, starting with the upper plane and, on each plane, the three levels of support are used that, in this case, correspond to the elementary actions which compose the problem: the “action of similarity” (identification of the difference between the figures), the “action of corresponding pictures search” (the grouping of similar figures), and the “action of exclusion which differs from the others” (elimination of the different figure to maintain the similarity of the others).

In the orienting scheme on the verbal plane, the first level offers less support; it only points to the beginning of the solution to the problem. If this level of support is not enough for the solution to the problem, the orientation is repeated and a little more support is offered, a little more substantial support in the orientation to find the answer. If that is still not enough, the third level of support is more complete and includes not only the general orientations to find the solution, but the logical principle for its solution. In each of the levels of support, the experimenter repeats the previous orientation and introduces a new one, a little more incisive in the building of its response, up to the third level, in which the child, besides receiving all the explanations, also has access to the logic of the solution.

Verifying the PDZ on the logical-verbal plane thus includes the solution to two problems at the logical-verbal level, with an aim to avoid a possible aleatory correct response to the problem. Once both problems are solved, the student is exempted from the experiment. If a problem is not solved, it is presented with concrete images.

Instructions and answers are the same as for the scheme of orientation on the logical-verbal plane; departing from the three levels of support which goes from the least to the most complete, according to the responses of the student. Verifying the PDZ on the plane of concrete images was confirmed if, in the end, the child responds to the two problems; if this does not happen, the presentation of OBA at the concrete level proceeds.

This latter is accomplished, in turn, with the use and the direct manipulation of objects, receiving one, two or three levels of support, depending on the need demonstrated by the child. The PDZ on the concrete action plane is confirmed when, from the scheme of orienting with use of concrete objects, the child solves two problems independently. If this does not happen, it is evident that this type of action is not in the child’s possible development zone.

These two series of the experiment describe the movement of the diagnostic which goes from the upper plane (logical-verbal) to the lower plane (concrete). It is important to recall, however, that the gradual formation of mental actions and concepts, in the educational and/or formative experimental of actions and concepts, follows a similar methodology, but from the lower plane (concrete) to the upper plane (logical-formal).

Experimental studies led to some conclusions which were, above all, important to the building of Galperin’s theory and method: “a) Actions are formed along with sensory images and concepts about objects of such actions, images and concepts represent different aspects of the same process ... ; b) The mental plane is just one of the ideal planes. The other is the perceptive plane. It is possible that a third plane, independent of the activity of each person, is constituted by the plane of language. The mental plane is formed only on the basis of the verbal form of action; c) Action moves to the ideal plane, be it completely, or just in its orienting part ... ; d) Transference of action in the ideal plane, particularly the mental plane, reflects its objective content through each of these planes and is expressed by multiple variations, successive, of the form of action; e) Transference of action to the mental plane, its internalization, is just one aspect of its variation. Other aspects not less important are the variations of all connections of the action, of the measure of its differentiation, of the measure of its domain, of the time, of the rhythm and the indicators of force” (Galperin, 1969 [2001f], pp. 69–70).

To sum up, theoretical investigation of the study of the genesis of the cognitive processes permits us to affirm that the gradual formation experiment is also a formative procedure of cognitive processes, and the difference between the diagnosis and the formation of mental actions and concepts is just in the direction of the experimental movement.

## Conclusion

The present study of the experimental method developed by Galperin, in an intense collaborative work, confirms that his theory was built on the basis of simultaneous production of *a study method of the genesis of the cognitive process and a theory of stage-by-stage formation of mental actions and concepts.* This reinforces the pertinence of the experiment designation of gradual formation which we have assumed for Galperin’s method.

We conclude that the OBA is an important instrument for forming new actions and concepts. Its elaboration is part of developmental learning processes in Galperin’s perspective and it is the nuclear cell of the study method and the learning method proposed by the author and his collaborators.

Since orientation and interventions in actions may be understood as the collaboration processes which occur in the student’s PDZ, a Vygotskyan reading of Galperin’s theory includes observation of the teacher’s role in the three meanings presented by Vygotsky (2003, 2007): as organizer, guide, and collaborator in the activity of students’ learning-development. Such adult–child collaboration, based on the teacher’s orientation and organization, are the methodological tools of formation and development as a gradual educational process by its nature of “gradual formation,” taking into consideration Galperin’s studies, which leads us to reaffirm the conclusion that his study method deserves the designation of “gradual formation experiment.”

Advances that the works of P.Ya. Galperin have brought to psychology as well as education have been an object of growing interest of psychologists and educators (Alvarez & Solovieva, 2019; Arievitch & Haenen, 2005; Haenen, 1988, 1993; León, 2019; Longarezi, 2021a, 2021b; Longarezi & Puentes, 2013, 2017; Mendoza & Delgado, 2018; Núñez, León, & Ramalho, 2020; Núñez, Pinheiro, & Gonçalves, 2019; Núñez & Ramalho, 2017, 2018; Puentes, Puentes, Araújo, & Claudino, 2018; Solovieva, 2014a, 2014b, 2014c; Solovieva & Rojas, 2013, 2020), who have put his theory and method on the scientific agenda and developed it in the scope of developmental theory in different countries. In this scenario, the current article is presented with its focus on the experimental method developed by Galperin and his collaborators.

## Footnotes

1 Even though there is no consensus that the productions of P.Ya. Galperin and N.F. Talyzina are a didactic system, that idea was supported by a recent study which demonstrated the veracity of such understanding. Cf.: Puentes & Longarezi (2020).

2 Although the stages have gone through several terms and classifications, in the history of Galperin’s method and *the theory of stage-by-stage formation of mental actions and concepts*, in general, they are: 1. Motivational stage; 2. OBA stage; 3. Stage of the formation of materialized action; 4. Stage of formation of the action in the external verbal language; 5. Stage of formation of the action in the inner verbal language; and 6. Stage of mental action.

3 The Orienting Basis of an Action (OBA) consists of “the anticipated representation of the task, as well as the orientation system necessary to its fulfillment, [which] are part of a future plan of action, the basis for its guidance.” (Galperin, 1959 [2001c], p. 46). “In the OBA, the theoretical model of learning activity is expressed as an operation system which regulates and drives learning into the specific conditions. It refers to the correct and rational building of the executive part” (Núñez & Oliveira, 2013, p. 299). Gal’perin, in his text *Types of orientation and types of formation of actions and concepts* (Galperin, 1959 [2001e]), characterizes several types of OBA, according to different levels of support. According to the author, the types of orientation are reduced, fundamentally, to three: “If the subject cannot form a complete guiding image of the new action and the experimenter cannot help, this image is incomplete and one has obtained the first type of guiding. If the same experimenter shows him the complete orienting basis of an action and demands an intense investigation of such, one obtains the second type of guiding. Finally, if the subject builds a complete orienting image in an individual way, one obtains a third type of orientation in the task” (Galperin, 1959 [2001e], p. 41).

4 The term “support” is used in this text in the sense of collaboration, as discussed by L.S. Vygotsky.
